# Generating Artificial Sensor Data for the Comparison of Unsupervised Machine Learning Methods

**DOI:** 10.3390/s21072397

**Published:** 2021-03-30

**Authors:** Bernd Zimmering, Oliver Niggemann, Constanze Hasterok, Erik Pfannstiel, Dario Ramming, Julius Pfrommer

**Affiliations:** 1 Institute of Automation Technology, Helmut-Schmidt-University, 22043 Hamburg, Germany; oliver.niggemann@hsu-hh.de (O.N.); erik.pfannstiel@hsu-hh.de (E.P.); rammingd@hsu-hh.de (D.R.); 2 Fraunhofer Institute of Optronics, System Technologies and Image Exploitation IOSB, 76131 Karlsruhe, Germany; constanze.hasterok@iosb.fraunhofer.de (C.H.); julius.pfrommer@iosb.fraunhofer.de (J.P.)

**Keywords:** machine learning, artificial data, anomaly detection

## Abstract

In the field of Cyber-Physical Systems (CPS), there is a large number of machine learning methods, and their intrinsic hyper-parameters are hugely varied. Since no agreed-on datasets for CPS exist, developers of new algorithms are forced to define their own benchmarks. This leads to a large number of algorithms each claiming benefits over other approaches but lacking a fair comparison. To tackle this problem, this paper defines a novel model for a generation process of data, similar to that found in CPS. The model is based on well-understood system theory and allows many datasets with different characteristics in terms of complexity to be generated. The data will pave the way for a comparison of selected machine learning methods in the exemplary field of unsupervised learning. Based on the synthetic CPS data, the data generation process is evaluated by analyzing the performance of the methods of the Self-Organizing Map, One-Class Support Vector Machine and Long Short-Term Memory Neural Net in anomaly detection.

## 1. Introduction

Data analysis and Machine Learning (ML) are major topics in Cyber-Physical Systems (CPS) due to their great potential for monitoring and optimization by the exploitation of acquired data. The development of ML methods that are suitable for CPS is an active field of research as these methods can be used to analyze a CPS (self-diagnosis), to ease system modifications (self-configuration) or to optimize the system’s performance (self-optimization). Here, the main challenge is choosing the ML methods that suit the data as well as the CPS characteristics. Since a wide variety of machine learning methods exists and they are used in several domains (e.g., healthcare [1]), comparisons of algorithms for the above-mentioned CPS use cases are essential. In domains such as image classification, standard datasets (e.g., Fashion-MNIST [2]) provide a benchmark for comparing different ML methods and their implementations across the scientific world [3].

For CPS, open access to real world data is very limited, and often the data are very domain-specific. Furthermore, first-principle simulation models usually require a great deal of effort and are domain-specific and often confidential; thus, they are hardly suitable for generating data that can be generally accepted as benchmarks in the research community. Thus, comparisons and assessments of machine learning algorithms in the context of CPS are scarce and anecdotal. In a situation where the problem of machine learning is not the lack of algorithms but their abundance, the lack of their comparability is a crucial problem for the creation of a theory that maps ML methods to the CPS characteristics and data. Such a theory would be a step towards greater industrial acceptance of ML methods.

In this paper, a first step towards the development of a suitable theory for CPS machine learning is taken by introducing the main idea of a system theory-based model of a CPS data generation process. The following research questions are addressed:

*Research Question 1 (RQ 1):* Can we formalize a CPS data generation model?
*Research Question 2 (RQ 2):* Can we classify typical failures of CPS and insert them into the CPS data generation model?
*Research Question 3 (RQ 3):* Can we use the CPS data generation model to generate artificial datasets for acceptable and unacceptable scenarios?
*Research Question 4 (RQ 4):* Can we use the generated acceptable and unacceptable datasets to evaluate and compare typical CPS machine learning algorithms for anomaly detection? Do the results follow our intuition regarding the complexity of the generated data as well as the expected behavior of the algorithms?

The authors do not claim that the CPS data generation model introduced here will replace real data. However, the solution developed here fulfills the basic requirements for such models and allows for a first theoretic and empiric comparison of algorithms. Thus, it may serve as a crystallization point for future research in machine learning for CPS. All data generation programs and all data are published at https://www.hsu-hh.de/imb/en/projects/ArtDataGen, accessed on 30 March 2021; thus, in the long-term, an accepted set of datasets for this community can be established.

After a review of the state of the art in Section 2, the model of the CPS data generation process is described in Section 3. Based on the model, corresponding data are generated, allowing for an empirical comparison and evaluation of machine learning methods in Section 4. The model for the data generation process is evaluated in Section 5. The conclusions of this work are summarized in Section 6.

## 2. State of the Art

Generating datasets for the evaluation of ML methods in the context of CPS is a rather new field. [4] has applied ML to some simple artificial datasets. For rather specific ML questions, artificial data have been used in [5,6].

Machine learning for CPS comprises a set of very different methods; e.g., statistical methods and neural networks [7,8,9]. Learning models for continuous processes can be difficult; often, methods of differential equations [10] are combined with optimizing Kalman filtering and stochastic processes such as the Hidden Markov Model or Gaussian [11]. An example of a solution in which digital computing processes are analyzed in combination with analog physical processes is given in [12]. There are different ways of analyzing continuous signals, such as the continuous time Bayesian network (CTBN) [13,14] or hierarchical clustering [15]. Continuous signals can also be evaluated by working with time-dependent machines or neural networks [16]. The discretization of neural networks for sequential data by extracting finite state machines from second-order recurrent neural networks [17] is part of the work in [18]; this is used to extract knowledge in a symbolic form from different continuous models. A new algorithm for extracting automata from recurrent neural networks that works with long short-term memory networks is presented in [19].The discretization of complex data based on deep learning, and on the other hand based on multiple levels of feature extraction, is introduced in [20]; here, speech spectrograms are discretized using a deep auto encoder network. A similar deep belief network, which consists of multi-layered Restricted Boltzmann Machines, is used to extract binary codes from text files in [21].

## 3. A Model of CPS Data Generation

### 3.1. Characteristics of CPS Data

First, characteristics of CPS are given, from which requirements for data generation are derived. CPS usually comprise a large number of sensors, actuators, controllers and electrical components whose signals are stored in databases. The data encode the temporal evolution of a number of physical processes that take place within the system, can be correlated and can have boundary conditions and constraints. Often, several parameters within the data describe different aspects of the same process; e.g., during gas heating, the temperature and the pressure are monitored. Thus, the high-dimensional signal observation space emerges from a small-dimensional manifold in a latent space that is governed by physical laws.

*Requirement 1:* The data generation must differentiate between a non-observable latent space and a high-dimensional observation space.
*Requirement 2:* The data must be first sampled from a sub-space in latent space. The sub-space must capture data dependencies—dependencies which are due to the physical laws governing the CPS behavior.
  CPS are time-dependent and mode-dependent systems which comprise discrete signals as well as time and value-continuous signals. Often, discrete signals such as opening a valve or turning off a robot trigger mode-changes [22]; i.e., they abruptly change the system behavior. Continuous signals comprise, e.g., energy consumptions, gas flows, temperature changes, etc.
*Requirement 3:* The solution must create time-series in which consecutive samples are closely related to each other.
*Requirement 4:* The data must be hybrid; i.e., they must comprise both discrete (control) signals and continuous values. Some discrete control signals must trigger mode changes; i.e., they must cause a change in the system behavior.
  A machine learning algorithm should be able to learn the low-dimensional behavior of the data in a high-dimensional space and it should be robust against the mentioned mode-changes. Furthermore, a common use case for the learned models is the detection of abnormal process behavior. Thus, machine learning for CPS is an individual research field requiring individual solutions and justifying the development of its own benchmark datasets.
*Requirement 5:* The data generation process must be able to insert typical faults into the data.

### 3.2. Concept of the CPS Data Generation Model

**Figure 1:**
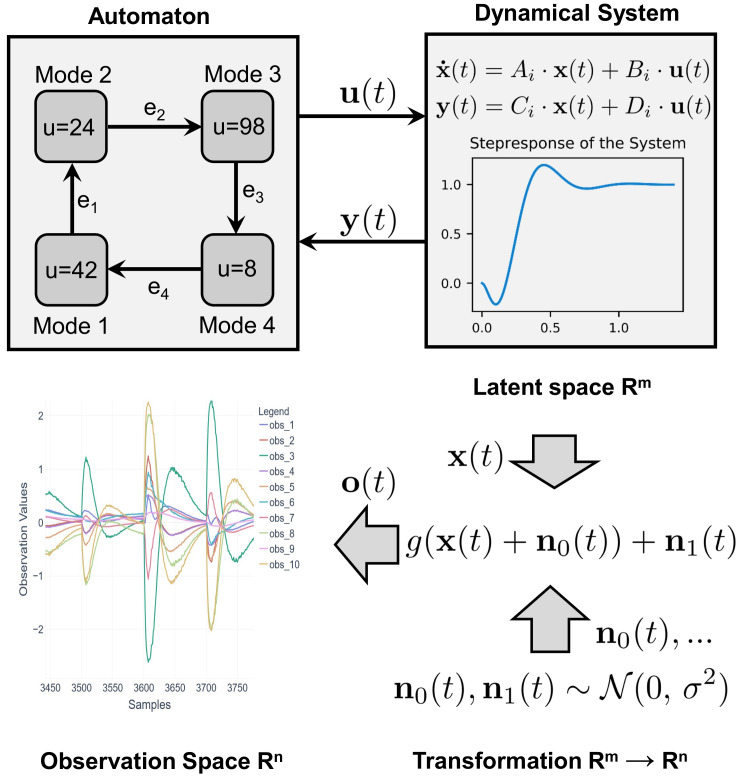
General concept of the CPS data generation model: An automaton (e.g., containing four modes $M_{i}$) interacts with a dynamical system. Its control values u(t) force the dynamical system to move its response y(t) towards the control values. While performing this movement, the internal states $x\left(t\right)\in R^{m}$, that define the order of the dynamical system change their values according to Equation (1). As the automaton cyclically evaluates whether $e_{i}$ from Equation (2) is true, mode changes are triggered automatically. The resulting trajectory of x(t) is then transformed trough the nonlinear mapping function g(·) (Equation (3)) into the observation space $o\left(t\right)\in R^{n}$.

Figure 1 sketches the main idea of the data generation process. The core idea is to have a non-observable behavior in an *m*-dimensional latent space that models the underlying physical behavior of the CPS (e.g., a thermodynamic process, where the (not directly measurable) dimensions *m* are {energy,entropy,ingredientcomposition}). The latent space is mapped into an observable (directly measurable) *n*-dimensional observation space (e.g., sensors measuring {temperature,pressure,mass,volume,flow}) where normally m≪n holds true.

In a latent space, the system behavior is modeled as a hybrid automaton [23]. Hybrid automata comprise *M* modes and M×E→M transitions; $E=\{e_{0},\ldots \}$ is a set of events. Normally, modes correspond to general system modes such as a “start-up phase”, while events correspond to control signals such as a “turn-on valve”. An automaton models the dynamical behavior in *m* latent dimensions by non-observable, state variables x(t) such as filling levels or momentum. Within each mode, the latent behavior is modeled by means of a linear Ordinary Differential Equation (ODE). Please note that different modes have different ODEs. The ODE for mode $M_{i}$ is defined as a state space model:

(1) $$\begin{array}{cc} \; & \dot{x}\left(t\right)=A_{i}\cdot x\left(t\right)+B_{i}\cdot u\left(t\right)\\ \; & y\left(t\right)=C_{i}\cdot x\left(t\right)+D_{i}\cdot u\left(t\right) \end{array}$$

with state variables $x\left(t\right)\in R^{m}$, system inputs $u\left(t\right)\in R^{p}$ and the system outputs $y\left(t\right)\in R^{q}$.

The matrices $A_{i},B_{i},C_{i},D_{i}$ from Equation (1) parameterize the behavior of the ODE in the following sense:

$A_{i}\in R^{m\times m}$ (System matrix): This models the dynamical behavior which can be characterized by its eigenvalues.
$B_{i}\in R^{m\times p}$ (Input matrix): This determines which state variables can be influenced by the input vector u(t)
$C_{i}\in R^{q\times m}$ (Output matrix): This maps the state variables x(t) to the output y(t).
$D_{i}\in R^{q\times p}$ (Feedthrough matrix): The system output is directly accessed by the system input (appears rarely in real-world systems)

The matrices are generated in a way that ensures the stability of behavior: the dynamics are defined by poles and zeros $p,z\in C^{m}$ of a transfer function G(s)—an approach typical for describing the I/O behavior of ODEs (more information can be found in [24]). Using the controllable canonical form for the ODE in Equation (1), the matrices $A_{i};B_{i};C_{i};D_{i}$ can be derived directly using the coefficients of G(s) [24]. Asymptotic stability is ensured by the real part of every pole $ℜ(p_{i})<0$ (a proof can be found in Chapter 3.2 of [25]). For matrices $A_{i};B_{i};C_{i};D_{i}$, as defined above, for t→∞, the limit y(t)=u(t) holds. For the sake of the comparability of y(t) and u(t), their dimensions are chosen to be q=p.

Modeling physical behavior by means of linear ODEs is a common-place and well-accepted approach [26]. For adding discrete (control) behavior to ODEs, hybrid automata are a well-established formalism [27,28]. Events $e_{i}$ trigger the transition from one mode $M_{i}$ to the next mode $M_{i+1}$. For the presented approach, we define events $e_{i}$ according to Equation (2).

(2) $$\begin{array}{cc} \; & e_{i}=\left(\left(y\left(t\right)\geq b_{l}\right)\vee \left(y\left(t\right)\leq b_{u}\right)\right)\wedge \left(|\dot{y}\left(t\right)|<\gamma \right)\\ \; & \mathrm{where}\;b_{l}=u_{i}-\epsilon \;\mathrm{and}\;b_{u}=u_{i}+\epsilon \;\epsilon ,\gamma \in R^{+} \end{array}$$

Using the commands $u_{i}$ of mode $M_{i}$ and some interval defined by ϵ, bounds $b_{l},b_{u}$ are generated. They are used to check if the dynamical system has executed the commands from the automaton’s mode. Furthermore, the parameter γ allows us to ensure that the event is triggered only if the system movements are below some threshold by the observation of $\dot{y}\left(t\right)$. To make the decisions non-deterministic to a certain extent, the time interval in Equation (2) is processed, which can be larger than the simulation time interval (e.g., a controller cyclically checks sensor values every 100 ms while the underlying process reacts more quickly). As CPS often show some cyclic behavior (i.e., a production system repeating a sequence of actions for every product), the automaton is able to model cyclic behavior by restarting at its initial mode (e.g., $e_{4}$ in Figure 1). Through environmental influences, these cycles also have a slightly unique character. Therefore, some noise $s_{u_{i}}$ is sampled from a normal distribution ~N(0,σ), which is the common case for a large number of environmental influences, and is added to the command vector $u_{i}$ of the modes $M_{i}$ of the automaton for every cycle.

**Figure 2:**
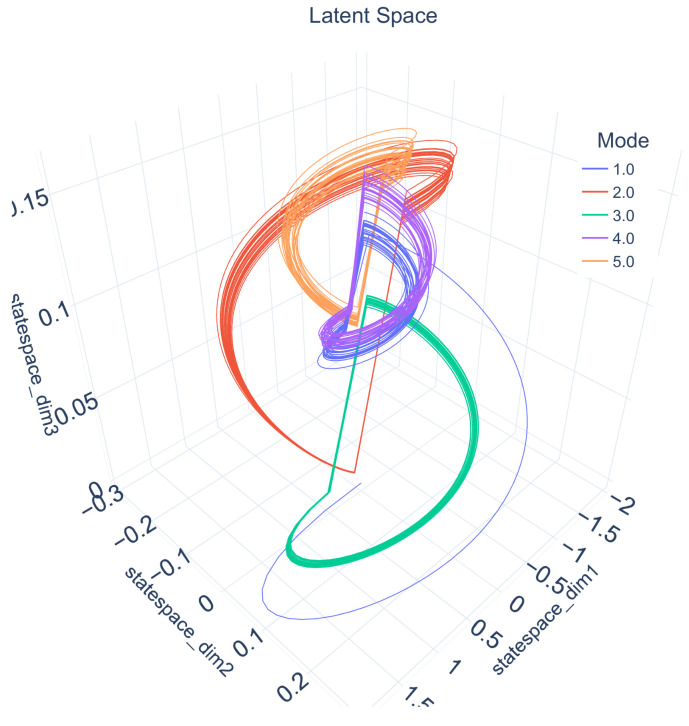
The latent space of $x\left(t\right)\in R^{3}$ of a third-order dynamical system over 25 cycles of a five-mode automaton. Every dimension of x(t) is shown as an axis and allows a graphical interpretation of the internal dynamics of the dynamical system from Equation (1). The colors indicate the trajectory taken to follow the commands within the automaton’s modes. It can be seen that each state has a unique shape.

To map from the *m*-dimensional, non-observable latent space to the *n*-dimensional observation space, the mapping function *g* is used (see also Figure 1). This latent space, including the system behavior, is shown in Figure 2. The mapping function *g* used here (see Equation (3)) is a superposition of linear, sinusoidal and exponential components:

(3) $$\begin{array}{cc} g(x(t)) & =\alpha_{1}\cdot \left(x\left(t\right)+n_{0}\right)E\\ \; & +\alpha_{2}\cdot \sin\left(x\left(t\right)+n_{1}\right)F\\ \; & +\alpha_{3}\cdot \exp\left(x\left(t\right)+n_{2}\right)G \end{array}\;$$

The function *g* is parameterized by matrices $E,F,G\in R^{m\times n}$ which are randomly taken at the beginning of the generation process. $n_{0},n_{1},n_{2}\sim N\left(0,\sigma \right)$ models added noise. $\alpha_{1},\alpha_{2},\alpha_{3}\in R$ weigh the different terms. E,F,G model interdependencies between signals. Sensor readings are modeled by the linear part. The sinoid term captures machine cycles. The exponential parts are typical when intensifying machine behavior; e.g., with reinforced errors. Based on this approach, a large number of feasible parameterizations and therefore datasets can be generated automatically.

### 3.3. Anomaly Insertion

CPS processes can deviate from their normal behavior due to wear or failures of system components; for example, the temperature of a gas may not increase due to a failure of heating, or the speed of a pump may decrease due to the abrasion of the ball bearings. The result is a deviation of the system state from the original trajectory which is propagated to the observation space. Anomalies that are only present in the observation space—e.g., caused by malfunctioning sensors—are beyond the scope of this paper.

We create abnormal datasets by the insertion of the following anomalies at different intensities. Please note that we use also cross combinations of them as more than one type of error can occur in a system:

*(i)* 
*Adding or removing modes from the automaton:* Adding modes corresponds to additional tasks or functions. Removing modes is often equivalent to skipping tasks.
*(ii)* 
*Changed noise:* The variance of the noise term $n_{i}$ is increased.
*(iii)* 
*Offset to u(t) or x(t):* Offset to the state vector of the mode’s ODE model; e.g., systematic errors.
*(iv)* 
*Shifting the poles p of the dynamical system:* The real part of the poles $ℜ(p_{i})$ determines how fast the system is able to move. By increasing (slower) or decreasing (faster), it can be simulated that, e.g., the masses of the system increase or decrease.

We differentiate between two types of methods for anomaly detection: (1) static methods that neglect temporal dependencies as they take the samples $x_{t}$ independently and (2) dynamic methods that incorporate temporal dependencies by processing pieces of a time series $x_{t-n},\ldots ,x_{t}$. While static methods aim to detect point anomalies (e.g., inserted anomalies (i), (ii) and (iii)), dynamic methods are able to detect contextual anomalies such as inserted anomaly (iv).

## 4. ML Methods

**Figure 3:**
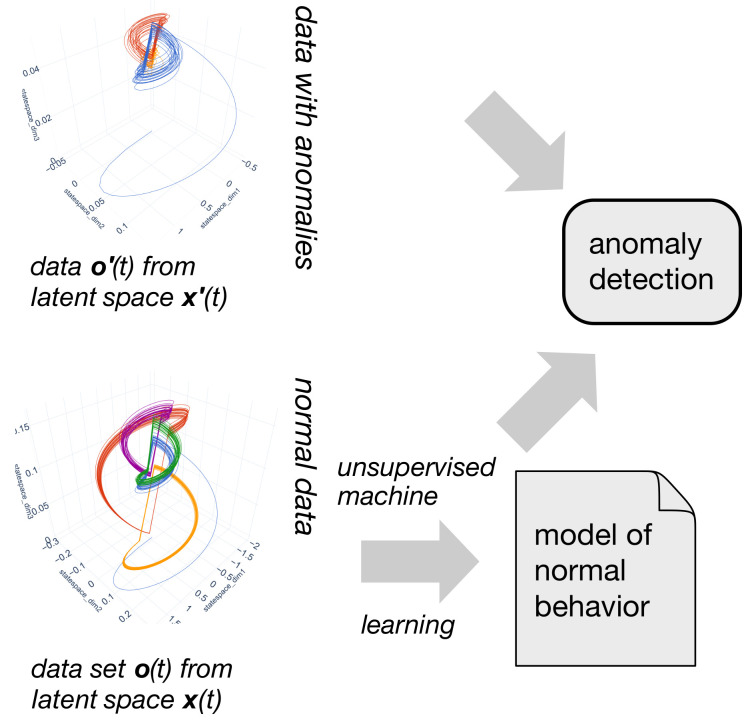
Using anomaly detection for the evaluation of unsupervised machine learning methods.

Learned models can be applied to several tasks in the context of CPS. In this work, we focus on anomaly detection using unsupervised machine learning. Figure 3 shows the main idea.

Self-Organizing Maps (SOMs), also known as Kohonen maps, were first proposed in 1982 [29] and have been widely applied in many fields since then. SOMs consist of neurons that are arranged on a two-dimensional grid and are associated with reference vectors in the observation space. The reference vectors are initialized randomly and then updated in a cycle. In each cycle—i.e., each iteration over the whole dataset—every data point $x_{n}$ is associated with the closest reference vector $z_{k}$, also denoted as the winning node. The winning node and all its neighboring neurons on the two-dimensional grid are then shifted towards the data point $x_{n}$ in the observation space. Both the size of the neighbor and strength of shift (learning rate) decrease with each cycle, resulting in the convergence of the map.

Support Vector Machines compute a classifier by maximizing the margin—i.e., the space—between the classes [30]. Using the so-called kernel trick—i.e., a mapping into higher dimensions—nonlinear-separable classes can also be classified. One-Class Support Vector Machines (1-SVMs) extend these algorithms to unsupervised learning [31]. For this, the margin between the class and the origin of the observation space is maximized. In this paper, a 1-SVM with a radial basis function kernel (RBF) is used for a static data analysis.

Long Short-Term Memory (LSTM) models have first been proposed as an alternative to the simple recurrent neural net by [32]. They are, in one form or another, a major part of state of the art algorithms in a wide area of domains ranging from speech, natural language and image recognition to deep reinforcement learning or time-series forecasting. In such use cases, they can be used to detect anomalies by comparing predictions with observations (see e.g., [33] applied to aircraft data, or [34], where LSTM is used to detect anomalies in different scenarios). LSTMs are used for dynamic data analysis. In this paper, they are used as a baseline for two dynamical anomaly detection approaches. In contrast to the methods mentioned above, they use $x_{t-n},\ldots ,x_{t}$ to predict the next values $x_{t+1}$. Furthermore, we employ a variant that uses an energy-based loss function, the Maximum Likelihood Estimation Loss function (MLE), as proposed by [9]:

(4) $$L_{t+1}=\sum_{i}\left[{\left(\frac{x_{t+1}-{\hat{x}}_{t+1}}{\sigma_{t+1}}\right)}^{2}+2\log\sigma_{t+1}\right]$$

It incorporates the standard deviation σ into the prediction and allows us to predict how certain (or uncertain) the prediction is [35]. For the LSTM algorithm, we employ the standard mean-squared-error (MSE) as well as the MLE approach, denoted as LSTM MSE and LSTM MLE.

## 5. Evaluation of Data Generation

### 5.1. Artificial Dataset

To evaluate the model presented, 1728 datasets of different complexity and error intensity were generated. For the sake of simplicity, the dynamical system for every mode $M_{i}$ remained the same and was of the third order (poles: −15,(−5+3j),(−5−3j); zeros 10); thus, our latent dimension was m=3. Furthermore, a scalar control value 0<u(t)≤100 for our experiments was used. The following main parameters were used and varied between the data sets:

- *$n_{\mathrm{modes}}$:* The automaton had {5;15} modes;
- *$n_{\mathrm{obs}}$:* The number of observation dimensions *n* was {10;50} sensors;
- The model was simulated over 25 cycles (no variation);
- $s_{u_{i}}$ Noise of different levels {2;5} was added;
- $\alpha_{2}$ and $\alpha_{3}$: Non-linear terms of Equation (3) were scaled by $\alpha_{2}=\left\{0;1\right\}$ and $\alpha_{3}=\left\{0;0.1\right\}$.

For the unacceptable datasets, the following errors were varied:

*(i)* 
Two modes were added and two modes removed;
*(ii)* 
Noise was added to the latent space ($n_{0}$ to $n_{2}$ of Equation (3) is increased);
*(iii)* 
Offset for $u_{i}$ of {5;15};
*(iv)* 
The poles shifted towards a faster ODE were {20%;50%}.

### 5.2. Evaluation Metrics for Anomaly Detection

Not all unsupervised ML methods provide a binary classification result. Some return a confidence or even probabilistic estimate that can be converted into a binary classification by the use of a threshold. However, he choice of the threshold is mostly arbitrary.

In anomaly detection tasks, the trade-off between false positives and false negatives depends on the use case. Usually, false negatives (no anomaly is detected when one is present) are penalized much more heavily than a false positive (a false warning). In order to make the evaluation independent of these application-specific trade-offs, we show receiver-operating-characteristic (ROC) curves—i.e., the true positive rate as a function of the false positive rate—to allow a comprehensive evaluation. In order to produce an absolute ordering of the anomaly detection algorithms, the area-under-the-ROC-curve (AUC) metric [36] is used. High AUC values indicate a good sensitivity to anomalous data and therefore a high efficiency for detecting anomalies. The AUC metric, however, assumes that true positives and false positives have equal importance.

### 5.3. Definition of Evaluation Criterions

To evaluate the suitability of artificial data is a challenge as no established definition of CPS data characteristics exist. Thus, to some extent, such an evaluation must be heuristic. In the following, we define six evaluation criteria on the behavior of ML methods to assess the data generation process and show that the artificial data capture features of real data recorded in CPS.

*Sensitivity to data complexity:* Each ML method should show a variance of results according to different difficulty levels in the data. Data complexity is defined in terms of the dimensions of the observation space, the number of modes and sinoid/exponential factors. Only if results vary according to characteristics such as the dimensions of the observation space, the count of modes and exponential factors can the performance of ML methods be analyzed.

*Improved anomaly detection for increasing pole shifts:* Increasing pole shifts should lead to better AUC results, especially for dynamical methods, as they are able to consider the timing behavior of the dynamical system (that is, changes in this case). Furthermore, MLE should outperform MSE optimization metrics as it in cooperates with uncertainty.

*Uncertain regions:* MLE-based methods should mark regions in the data where variance changes dynamically and/or the uncertainty in the data is non-negligible. This should be the case near the mode changes, as Equation (2) is evaluated more slowly than the simulation.

*Superior performance of SVM for large observation spaces:* SVM should perform especially well for high numbers of observation dimensions, as more observation dimensions from the same latent space should simplify the finding of suitable hyperplanes.

*Influence of non-linear effects:* The larger the influence of non-linear effects (e.g., exponential and sinoid terms in the mapping function *g*), the worse the ML methods perform with regard to anomaly detection.

*Consistent performance on real and artificial data:* The ML methods should show a similar behavior for real data to that for artificial data.

In the following, these evaluation criteria are analyzed based on the AUC score.

#### Sensitivity to Data Complexity

**Figure 4:**
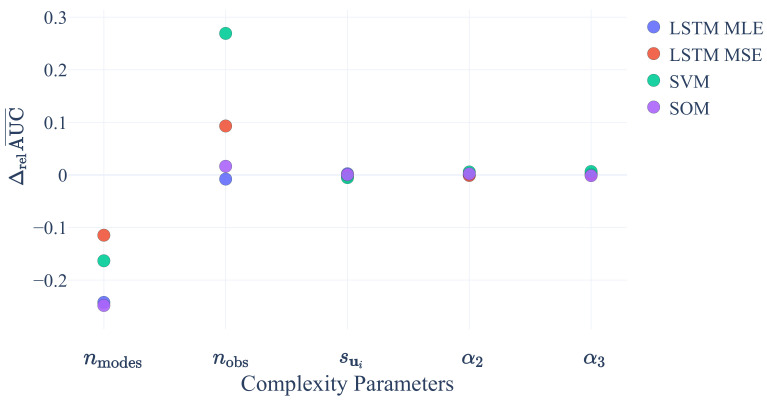
Relative difference of average area-under-the-curve (AUC) values $\Delta_{\mathrm{rel}}\bar{\mathrm{AUC}}$ (see text for exact definition) as a function of five parameters that are relevant for data complexity.

To determine the sensitivity of the ML algorithms to data complexity, we investigated the impact of variations in $n_{\mathrm{modes}}$, $n_{\mathrm{obs}}$, $s_{u_{i}}$, $\alpha_{2}$ and $\alpha_{3}$ on the AUC score. We averaged the AUC scores over all variations except that for the parameter under investigation. We denoted the average as $\bar{\mathrm{AUC}}$. Based on this, we computed the relative difference between the $\bar{\mathrm{AUC}}$ values from the two settings of the parameter under investigation and denoted the results as $\Delta_{\mathrm{rel}}\bar{\mathrm{AUC}}$. Figure 4 shows $\Delta_{\mathrm{rel}}\bar{\mathrm{AUC}}$ for the four ML algorithms as a function of the five data complexity parameters.

We conclude that variations in $s_{u_{i}}$, $\alpha_{2}$ and $\alpha_{3}$ have a minor impact on the AUC values below 1% for all ML algorithms. However, an increase in the number of modes from 5 to 15 results in a decrease in the AUC by 10% to 25%. As expected from intuition, a larger number of modes increases the difficulty of detecting anomalies. In contrast, increasing the number of dimensions in the observation space from 10 to 50 increases the AUC by about 27% for SVMs and about 10% for LSTM MSE. Thus, it becomes easier for those algorithms to detect anomalies if information from more dimensions becomes available. While SOMs still profit from an increase in dimensions, although on a low level of the order of ∼2%, the ability of the LSTM MLE algorithm to detect anomalies is decreased by about 1%. The cause of this is subject to future investigations.

### 5.4. Improved Anomaly Detection for Increasing Pole Shifts

**Figure 5:**
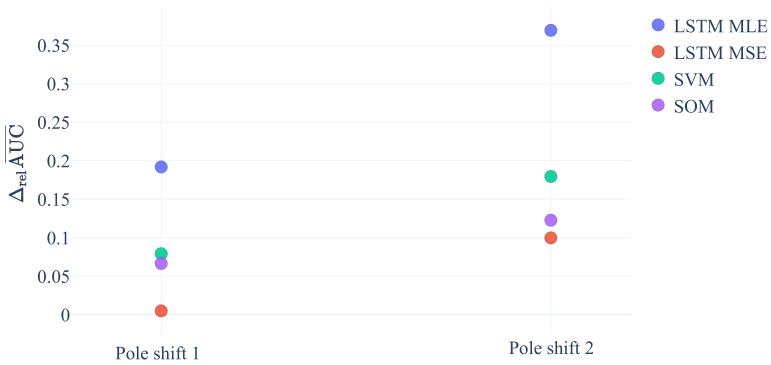
Comparison of Maximum Likelihood Estimation (MLE) versus mean-squared-error (MSE) as function of variations in the number of dimensions in the observation space and pole shift.

Figure 5 shows the previously defined $\Delta_{\mathrm{rel}}\bar{\mathrm{AUC}}$ values for variations in pole shifts. Pole shift 1 denotes the increase of the pole shift from 0% to 19%, and pole shift 2 denotes the increase from 10% to 37%. Since all values of $\Delta_{\mathrm{rel}}\bar{\mathrm{AUC}}$ are positive, we can conclude that an increase in pole shift generally improves the performance of all algorithms in detecting anomalies while LSTM MLE profits most. It follows our intuition that the LSTM MLE method performs well with this type of error as it is a dynamical method. Through the ability to learn a difference equation of the normal ODE, it can easily detect changed gradients in the unacceptable dataset.

### 5.5. Uncertain Regions

An important key insight is that the LSTM MLE algorithm allows it to be shown that the uncertainty behaves as expected from the data generation. Table 1 shows the MSE and uncertainty in the region of a state change from the automaton. The randomness caused by sampling the automaton more slowly than the simulation model (100 ms vs. 1 ms) was correctly reflected by a significantly higher uncertainty estimate 10 ms before the mode changed.

Furthermore, noise in the automaton’s target values was correctly considered be a higher uncertainty estimate shortly after the mode changed. When considering the MSE, this kind of hidden uncertainty cannot be observed.

### 5.6. Superior Performance of SVM for Large Observation Spaces

**Figure 6:**
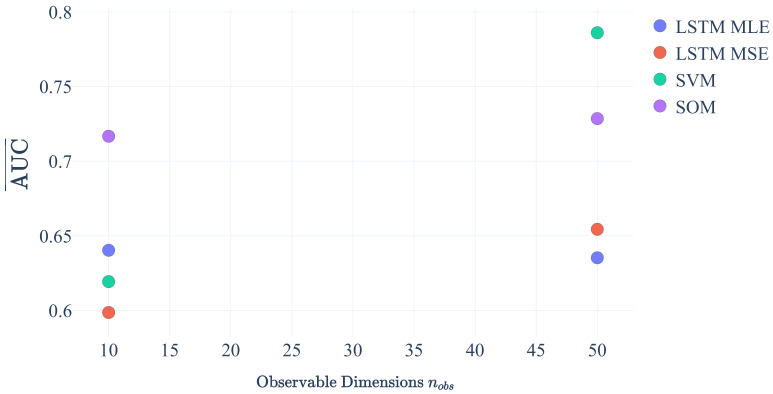
Average AUC for the four Machine Learning (ML) algorithms as function of the number of dimensions in the observation space.

Figure 6 shows that, for a large number of observation dimensions, SVM outperforms the other methods. This was expected as more observation dimensions make it easier for the SVM to separate the modes via its hyper planes.

### 5.7. Influence of Non-Linear Effects

**Figure 7:**
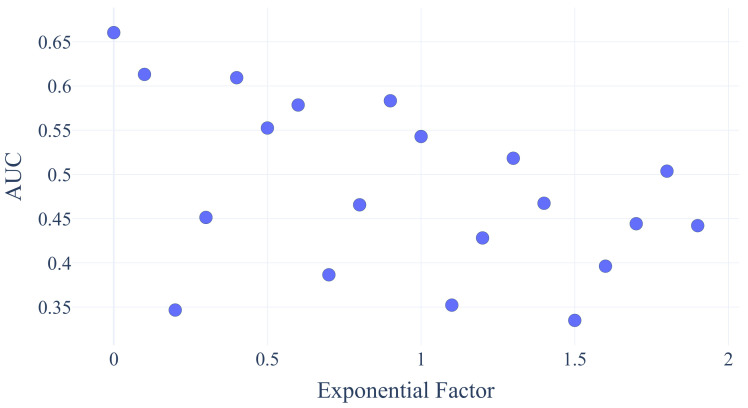
AUC receiver operating characteristic (ROC) values for a decreasing number of rising exponential factors.

Figure 7 shows that the performance of the LSTM MLE Method decreases as the non-linearity increases through the exponential factor $\alpha_{3}$ in Equation (3). Future work will investigate why some of the results are below an AUC of 0.5.

### 5.8. Consistent Performance on Real and Artificial Data

**Figure 8:**
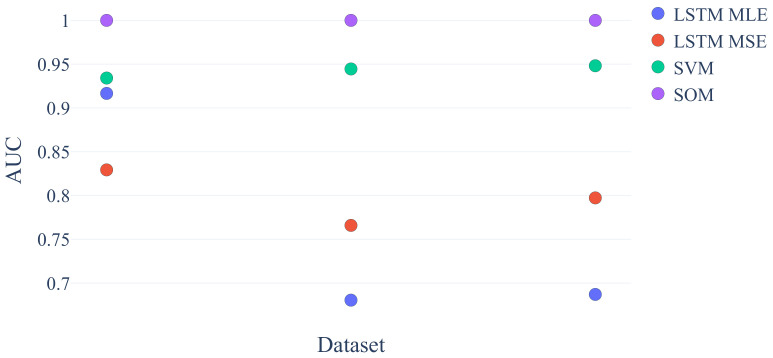
AUC ROC values for real world dataset.

Figure 8 shows that the dynamic (LSTM) as well as the static methods (SVM) are able to perform well on a real dataset from a Vega shrink-wrapper [37]. The dataset consists of three datasets from seven sensors (observation dimensions). In case of an anomaly, the cutting blade was worn out.

## 6. Conclusions

We have introduced a theoretical system-based model for the generation of artificial data for CPS, consisting of a hybrid automaton that uses an ODE to generate a contentious latent space (*RQ 1*). With the aim of anomaly detection, four types of typical CPS failures were defined and could be inserted into the CPS data generation model (*RQ 2*). Furthermore, we generated 1728 datasets containing training, acceptable and unacceptable data. Each dataset had a different complexity and error intensity (for the unacceptable case) (*RQ 3*). Using these datasets, we compared four ML methods (SOM, 1-SVM, LSTM MLE and LSTM MSE) with regard to five intuitive evaluation criterions. The results show that the data generation process produced data and corresponding ML results which were consistent with theory and experiences; i.e., the generated datasets resembled, from an ML point-of-view, real data (*RQ 4*).

We conclude that the generated data can be used to benchmark algorithms, check the correctness of new implementations and compare algorithms’ results. In particular, the implementations for the SOM and 1-SVM show comparable results; i.e., the results support the correctness of both implementations. The artificial data can also be used to analyze systematically the influence of individual data characteristics on the results of specific ML algorithms. For example, 1-SVM is less sensitive to changes of the observation space dimensions than SOM, which is probably due to the kernel trick and is therefore consistent with the theory.

As only some exemplary ML methods have been chosen to evaluate the data generation our work, this gives a limited picture for the creation of a benchmark of the methods. This will be investigated in our future work, as we will continuously add further benchmark algorithms and improve the data generation method. Furthermore, more elaborate comparisons of the behaviors of the anomaly detection methods are planned. An open question is why a static method (e.g., SVM) outperforms dynamical methods (e.g., LSTM).

For further analyses, comparisons and validations, the authors will publish all data, the complete data generation code and the used algorithm implementations. Additionally, the code for a systematic comparison of ML methods will be published in the future (https://www.hsu-hh.de/imb/en/projects/ArtDataGen, accessed on 30 March 2021).

## Figures and Tables

**Table 1:** Exemplary comparison of MSE to MLE in the area of a change in the mode of the automation for a single observation variable.

	σ (MLE)	MSE
Mean value dataset	0.057	$3.88\cdot 10^{-3}$
10 ms before mode change	0.099	$0.487\cdot 10^{-3}$
5 ms after mode change	0.2	$1.2\cdot 10^{-3}$

## Data Availability

The data presented in this study are openly available in https://www.hsu-hh.de/imb/en/projects/ArtDataGen.
